# A* Sargassum fluitans* Borgesen Ethanol Extract Exhibits a Hepatoprotective Effect* In Vivo* in Acute and Chronic Liver Damage Models

**DOI:** 10.1155/2018/6921845

**Published:** 2018-12-20

**Authors:** Carlos Quintal-Novelo, Jorge Rangel-Méndez, Ángel Ortiz-Tello, Manlio Graniel-Sabido, Rebeca Pérez-Cabeza de Vaca, Rosa Moo-Puc

**Affiliations:** ^1^Unidad Médica de Alta Especialidad, Centro Médico “Ignacio García Téllez”, Instituto Mexicano del Seguro Social, Calle 41 No. 439, Col. Industrial, 97150 Mérida, Yucatán, Mexico; ^2^Unidad de Investigación Médica, Centro Médico “Ignacio García Téllez”, Instituto Mexicano del Seguro Social, Calle 41 No. 439, Col. Industrial, 97150 Mérida, Yucatán, Mexico; ^3^Laboratorio de Espectrometria de Masas, Facultad de Química, Universidad Autónoma de Yucatán, Calle 43 No. 613 x Calle 90, Col. Inalámbrica, 97069 Merida, Yucatan, Mexico; ^4^Instituto de Fisiología Celular, UNAM, Departamento de Biología Celular y Desarrollo, Laboratorio 305-Sur, Circuito Exterior s/n Ciudad Universitaria, Del. Coyoacán, 04510 Mexico City, Mexico; ^5^Research Coordination, Centro Médico Nacional “20 de Noviembre”, ISSSTE, Mexico City, Mexico

## Abstract

One of the leading causes of death worldwide, cirrhosis, is a liver condition characterized by chronic necrosis, inflammation, and fibrosis. Hepatoprotective compounds, such as antioxidants, can prevent fibrosis. Macroalgae (seaweed) contain high amounts of antioxidant compounds and are plentiful; indeed, species such as* Sargassum fluitans *Borgesen (Phaeophyceae) carpet many beaches in the Caribbean Basin. An* in vivo* assay was done evaluating the possible hepatoprotective effect of a* Sargassum fluitans *ethanol extract. Two murine liver damage models were employed: acetaminophen (APAP) in Balb/c mice to induce acute damage; carbon tetrachloride (CCl_4_) in Wistar rats to induce chronic damage. Serum liver enzyme levels and relative liver weight were measured, and histopathological and immunohistochemical analyses of liver tissue sections were done. Both APAP and CCl_4_ significantly raised serum enzyme marker enzymes. Administration of 50 mg/kg* S. Fluitans *ethanol extract reduced this APAP- and CCl_4_-induced elevation to normal levels. This effect was corroborated by the extract's inhibition of inflammation and fibrosis in liver tissue observed in the histopathological analysis. The analyzed* S. fluitans *ethanol extract exhibited an* in vivo* hepatoprotective effect in acute and chronic liver injury models.

## 1. Introduction

Liver injury of many kinds can cause necrosis, inflammation, fibrogenesis, and eventually cirrhosis. This condition is histologically characterized by diffuse nodular regeneration surrounded by dense fibrotic septa with subsequent parenchymal extinction and collapse of liver structures; the end result is pronounced distortion of hepatic vascular architecture [1]. The World Health Organization (WHO) reports that approximately 2.3 million people die of liver diseases every year, of which 16.6% are attributed to substance abuse [2].

Colchicine is the treatment currently used to inhibit hepatocellular secretion of collagen and stop fibrotic tissue production. Its efficacy has not been yet confirmed due to its severe adverse effects [3, 4]. The lack of an effective therapy to prevent progression of liver damage has generated several lines of research including the search for compounds with hepatoprotective activity. These are intended to protect the liver from damage caused by ingested toxins, decrease alterations caused by free radicals, and prevent cirrhosis [5].

Reactive oxygen species play an important role in initiating hepatic fibrogenesis, a process characterized by excessive collagen deposition in the extracellular matrix in response to activation of hepatic stellate cells. Reactive oxygen species (ROS) cause lipid peroxidation and necroapoptosis of hepatocytes, amplify the inflammatory response, stimulate production of profibrogenic mediators from Kupffer cells, and recruit circulating inflammatory cells [6]. New molecular targets have been identified through an understanding of the mechanisms involved in liver fibrosis. Molecules with antioxidant, antifibrotic, and/or anti-inflammatory activities can exercise a hepatoprotective effect on liver tissue [7].

Plants are a primary focus in the search for hepatoprotective compounds and have yielded polyphenols such as silymarin. Marine resources constitute another promising source and studies have been done on carotenoids, fucoidans, phenolics, and chlorogenic compounds from sea cucumber and macroalgae species [8]. The latter have received particular attention as a potential source of hepatoprotective drugs because they exhibit broad structural diversity induced by the ecological pressures they experience. They produce myriad secondary metabolites that function as antioxidants, and provide protection against viral diseases, pathogenic fungi, and predators, among other functions [9].

Tropical macroalgae species are known to produce antioxidant compounds such as ascorbic acid. Many have been studied for their polyphenols content, including* Dictyota cervicornis, D. ciliolata, D. crenulata, Lobophora variegata, Turbinaria tricostata, Padina gymnospora, Sargassum pteropleuron, *and* Sargassum ramifolium *[10]. An extract from the macroalgae* Sargassum fluitans* is reported to have* in vitro *antioxidant and protective effects on hydrogen peroxide-induced damage in a human liver cancer cell line [11].

*Sargassum fluitans *Borgesen is a brown seaweed belonging to the order Fucales and is distributed along the Caribbean coast of the Yucatan Peninsula (Mexico) [12]. Indeed, this seaweed species is so plentiful that it can pose ecological problems as massive rafts of it and other seaweeds regularly wash up on beaches, negatively affecting regional marine life and damaging the area's tourism industry and economy. Since a* S. fluitans* extract is known to exercise antioxidant and protective effects on live cells* in vitro*, the present study objective was to evaluate* in vivo* the hepatoprotective potential of a* S. fluitans *ethanol extract in acute and chronic models of liver damage.

## 2. Materials and Methods

### 2.1. Chemicals

Carbon tetrachloride (CCl_4_), acetyl-para-aminophenol (APAP), N-acetylcysteine (NAC), and silymarin (Sil) were purchased from Sigma-Aldrich (St. Louis, MO, USA).

### 2.2. Algal Material

*Sargassum fluitans *Borgesen (Fucales, Sargassaceae) was collected on the Caribbean coast of the Yucatan Peninsula at Puerto Morelos, Quintana Roo, Mexico, during winter 2016 (20°50′43.5′′N; 86°52′36.4′′W).Voucher specimens were collected and identified. The collected seaweed was washed with freshwater to remove excess seawater, stored in plastic bags, and kept on ice during transport. In the laboratory it was washed again with freshwater to remove salts, sand, and epiphytes and stored at -20°C until processing.

### 2.3. Extraction Procedures

Extracts were produced by milling* S. fluitans *(10.9 kg) with 80% v/v ethanol and extracting by maceration at room temperature for 12 h. The remaining vegetal material was again extracted with 80% v/v ethanol at 70°C for 12 h by reflux. Both extracts were pooled and then concentrated under reduced pressure and lyophilized.

### 2.4. High Performance Liquid Chromatography

High performance liquid chromatography (HPLC) was done using an Agilent 1260 liquid chromatography system fitted with a quaternary pump, autosampler, and diode array detector set at 245 nm. A Hypersil GOLDC18 column (Thermo) (150 x 4.6 mm, 5 *μ*m) was used coupled to an analytical guard column (Zorbax Extend C18, Agilent, 4.6 x 12.6 mm, 5 *μ*m) and operated at a column temperature of 42°C. The mobile phase consisted of (A)1% (v/v) acetic acid and (B) methanol at a 1.2 mL/min flow rate, followed by a linear elution gradient: initial, 99% A for 2 min; decrease to 25% A over 18 min and hold for 5 min; increase to 99% B over 4 min and hold for 3 min; returned to 99% A for 5 min; and a final reequilibration of 99% A for 10 min before the next injection. Sample injection volume was 20 *μ*L (4.50mg/mL).

#### 2.4.1. Mass Spectrometry

This analysis was done with a HPLC Agilent 1290 liquid chromatography system fitted with a binary pump, autosampler, and mass spectrometer with an ESI interface and a QQQ mass analyzer. Chromatographic conditions were as described in the previous section. Gas temperature was 300°C, gas flow 5 L/min, nebulizer 45 psi, sheath gas temp 250°C, and sheath gas flow 11 L/min. Capillary voltage was 3500 V and nozzle voltage was 500 V. The mass spectrum was recorded within a range of* m/z* 70-1,000.

### 2.5. Toxicity Assay

Maximum tolerable dose was determined using BALB/c strain mice (8-10 weeks of age; 20-25 g weight), with three mice per treatment [13]. The fixed dose method was employed [14], which consists of administration of the highest EE dose (5 g/kg) by gavage to a group of three mice following the diagram proposed by the Organization for Economic Cooperation and Development [15]. For fourteen days after administration any signs of toxicity or death were recorded. If this occurred, a lower dose was administered and the animals were again observed for 14 days. This procedure was repeated until no signs of toxicity and death were observed, thus establishing the EE's maximum tolerable dose [15].

### 2.6. Acute Hepatoprotective Model

The animals were randomly divided into five groups (n= 4 mice per group): control group (4 mL/kg vehicle); EE group (50 mg/kg* S. fluitans* EE); APAP group (vehicle + 250 mg/kg APAP); NAC + APAP group (50 mg/kg N-acetylcysteine + 250 mg/kg APAP); and EE + APAP group (50 mg/kg EE + 250 mg/kg APAP). Before beginning the assay, the mice were fasted for 10 h and weighed. They were then intragastrically administered the corresponding dosage of treatment or vehicle, and the APAP or vehicle was injected intraperitoneally in an equal volume of 0.9% sodium chloride (NaCl) solution. After 24 h, the mice were killed by CO_2_ inhalation and blood samples taken by intracardiac puncture to quantify serum biochemical markers. The liver was extracted and weighed to calculate relative liver weight (RLW). The blood samples were centrifuged at 3000 rpm for 20 min (4°C) and the supernatant was stored at -20°C until biochemical analysis.

### 2.7. Chronic Hepatoprotective Model

Hepatic fibrosis was induced in a rat model by subcutaneous injection of CCl_4_ (0.60 mL/kg body weight) in 25% corn oil injected twice weekly for twelve weeks. Silymarin (Sil) and EE were prepared in distilled water and administered once daily to each animal by oral gavage one week prior to the beginning of CCl_4_ administration and during the twelve weeks thereafter. The rats were randomly divided into five groups (n=6) and treated simultaneously with one of the following treatments: control group (corn oil vehicle + distilled water vehicle); EE group (corn oil vehicle +50 mg/kg EE); CCl_4_ group (CCl_4_+ distilled water vehicle); Sil+ CCl_4_ group (CCl_4_+ 100 mg/kg silymarin); and EE +CCl_4_ group (CCl_4_ + 50 mg/kg EE). Body weight (BW) was monitored weekly. After treatment conclusion, all animals were assayed for sleeping time (see below).Twenty-four hours after conclusion the animals were killed by CO_2_ inhalation, blood samples collected, and liver lobes immediately excised, weighed, and preserved for histological analysis.

### 2.8. Biochemical Markers

Serum levels of alanine aminotransferase (ALT), aspartate aminotransferase (AST), and alkaline phosphatase (AP) activities were measured, as was the serum albumin content (ALB). These were quantified by an automatic analyzer (VITROS System Integrated 5600; Ortho Clinic Diagnostic) at the Ignacio Garcia Tellez National Medical Center Clinical Laboratory of the Mexican Institute of Social Security (IMSS).

### 2.9. Sleeping Time

Liver functionality was evaluated with the sleep induction test. Pentobarbital (20 mg/kg) was intraperitoneally injected and sleeping time measured; maximum measured elapsed time was 60 min [16, 17].Time was recorded at injection and when the rat could no longer accomplish the righting reflex (i.e., the ability to right itself), it was then placed on its back in a standard cage, and time was recorded again when the righting reflex was regained. Sleeping time was the time from loss of righting reflex to its return [18].

### 2.10. Histopathological Analysis

Liver samples were fixed in 10% paraformaldehyde in 0.1% phosphate buffer saline (PBS) and embedded in paraffin. Sections (4-5 *μ*m thick) were prepared using hematoxylin-eosin (H&E) or Masson's trichrome (MT) stains. Histological assessments of hepatocyte degeneration and fibrosis were done using these H&E- and MT-stained sections. Images were taken with a high-resolution video camera (Canon PC1089) connected to a light microscope (Axiostar Plus Carl Zeiss) at 40X magnification. These were taken by the same person in a blinded manner. Quantitative fibrosis was analyzed using the US National Institutes of Health (NIH) ImageJ Analysis Program. Blue (collagen) staining was normalized against red (hepatocyte-parenchyma) staining for each liver. The fibrosis was calculated as a percentage of total hepatic area and expressed as the average of five randomly selected tissue sections from each liver [19].

### 2.11. Immunohistochemical Analysis

Formalin-fixed, paraffin-embedded (FFPE) liver tissue samples were sectioned (4-5 *μ*m) with a microtome. Sections were mounted on glass slides, coated with poly-L-lysine solution, and microwaved in preparation for deparaffinization. Xylene-ethanol deparaffinization and rehydration were followed by citrate-buffer antigen retrieval. Immune staining antibodies were diluted at a 1:50 TGF*β*1 antibody (TB21): sc-52893 ratio and viewed in a Dako® HRP-DAB system. Images were taken with a high-resolution video camera (Canon PC1089) connected to a light microscope (Axiostar Plus Carl Zeiss) at 40X magnification. These were taken by the same person in a blinded manner. Quantitative fibrosis was analyzed using the US National Institutes of Health (NIH) ImageJ Analysis Program.

### 2.12. Statistics

Results were reported as the mean ± standard deviation. A one-way analysis of variance (ANOVA) was run with Tukey post hoc tests and a* p* ≤ 0.001 confidence level. Statistical analyses were done with the GraphPad Prism ver. 5 statistical software program.

## 3. Results and Discussion

A* Sargassum fluitans* ethanol extract has been shown to exhibit a protective effect* in vitro *against oxidative stress in HepG2 cells [11]. The present study follows up on this previous report by evaluating the potential hepatoprotective effect of* S. fluitans *ethanol extract in acute and chronic models of liver damage.

### 3.1. HPLC Profile

**Figure 1:**
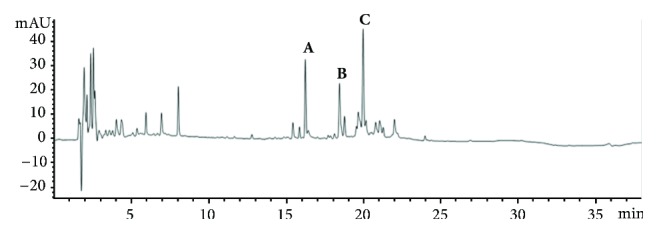
HPLC chromatogram of* S. fluitans* ethanol extract at 245 nm. Four major peaks were identified: A (retention time 8.01 min); B (retention time 16.21 min); C (retention time 18.42 min); and D (retention time: 19.90 min).

Ethanol extract yield was 64.5 g (3.70%; dry weight). A HPLC analysis identified three major resolved peaks: A (RT: 8.01 min), B (RT: 16.22 min), C (RT: 18.42 min), and D (RT: 19.92 min) (Figure 1). These peaks matched previous reports of phenolic compounds known as phlorotannins [20–22]. The peak at retention time 16.21 min exhibited an [M-H]^+^ ion at* m/z *219 which corresponded to a phlorotannin dimer. The peak at retention time 18.42 min showed an [M-H]^+^ ion at* m/z *247, an isomeric phlorotannin dimer. The peak at 19.90 min showed an [M-H]^+^ ion at* m/z *353, a phlorotannin trimer (Supplementary Figure S1). These results agree with previous reports of phenolic compounds content in ethanol extracts of* Sargassum *species [22–24].

### 3.2. Toxicity Assay

Maximum tolerable dose of the EE was 5 g/kg, since no acute toxicity or death was observed at this level (data not shown). This is the first* in vivo* assay of the toxicity of a* S. fluitans *ethanol extract, and the results agreed with previous studies of extracts from* Sargassum *species. Firdaus et al. (2012) evaluated the toxicity of a* Sargassum echinocarpum *methanol extract in mice at 5 g/kg with no observed toxicity [25]. Wariz et al. (2016) orally administered a* Sargassum* sp. methanol extract to mice at increasing doses (0.5-2 g/kg body weight) with no observed toxicity [26].

### 3.3. In Vivo Acute Hepatoprotective Assay

Evaluation of any hepatoprotective effect of the EE was done with an acute damage test involving a toxic dose of APAP in mice and recording relative liver weight (RLW) and AST and ALT enzyme activities (Table 1). The APAP group exhibited a significant increase in RLW versus the control, indicating APAP-induced inflammation. In contrast, RLW in the EE + APAP group was significantly lower than in the APAP group, suggesting that EE affords protection against acute APAP-induced liver injury. No differences were present between the control, EE, NAC + APAP, and EE + APAP groups (Table 1).

Liver enzyme activities in the control group were normal (ALT = 44.75 U/L; AST =68.25 U/L); this was considered as baseline (100%) activity in the absence of APAP injury. Activities in the EE group did not differ from the control group. However, in the APAP group ALT activity increased fourfold over the baseline and AST activity sevenfold (*p*<0.001).In the NAC + APAP group ALT and AST activities increased twofold (p<0.001), and in the EE + APAP group these activities increased 1.7 times above levels in the control group (*p*<0.001).

These results generally coincide with a series of previous studies. Vázquez R. et al. (2012) showed that a* Sargassum siliquosum *aqueous extract lowered APAP-induced AST and ALT activities in Sprague-Dawley rats [27]. In another study, Khan H. et al. (2016) evaluated* Sargassum variegatum*,* Sargassum terrarium*, and* Sargassum binderi *ethanol extracts in an* in vivo* model with APAP-CCl_4_-induced damage, reporting that the* S. variegatum *extract exhibited the highest hepatoprotective activity [28]. Raghavendran et al. (2004) reported that a* Sargassum polycystum* extract has a hepatoprotective effect on APAP-induced liver damage in rats since AST and ALT activities decreased compared to the positive control. The apparent acute hepatoprotective effect of the* S. fluitans *EE suggested the need to evaluate its hepatoprotective effect in a chronic liver damage model [29].

### 3.4. In Vivo Chronic Hepatoprotective Assay

Carbon tetrachloride (CCl_4_) is commonly used as a model to study hepatotoxicity [30]. A chronic model of hepatic fibrosis was produced by administering CCl_4_ (0.60 mL/kg) to rats for twelve weeks. After sleeping time was analyzed, the rats were euthanized and blood and liver samples taken to quantify serum liver enzymes (AST, ALT, AP, and ALB) and RLW. The liver tissues were analyzed with H&E stain to measure hepatocyte degeneration and MT stain to determine degree of fibrosis, and immunohistochemistry was applied to quantify TGF*β*1 expression.

#### 3.4.1. Sleeping Time

**Figure 2:**
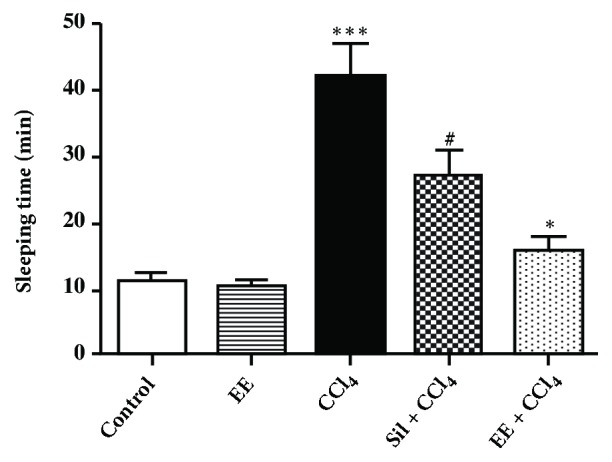
Effect of* S*.* fluitans *ethanol extract on sleeping time in the groups of a chronic liver damage model in rats.* p*-Values: *∗* < 0.001 vs. CCl_4_; ^#^ < 0.05 vs. CCl_4_; *∗∗* < 0.001 vs. control, according to one-way ANOVA with a Tukey post hoc test.

Sleeping time is the time rats remain asleep after a pentobarbital dose (20 mg/kg in this case). Control group sleeping time was 10.97 ± 3.10 min, which was considered the minimum sleeping time. The EE group did not differ from the control group. In the CCl_4_ group sleeping time was four times longer than in the control group (*p*<0.001).This was followed by a 2.37-fold increase in the EE + CCl_4_ group (*p*<0.05) and a 1.28-fold increase in the Sil + CCl_4_ group (*p*<0.001) (Figure 2).Pentobarbital-induced sleeping time duration is related to liver metabolism capacity. The enzyme cytochrome P450 (CYP) is responsible for drug biotransformation and hence for the conversion of CCl_4_ into the trichloromethyl radical (CCl_3_) that induces liver damage [30]. The P450 enzyme also transforms pentobarbital, allowing for its conjugation and metabolism in the liver [31]. Longer sleeping times indicate increased cellular damage in the liver; therefore the shorter times observed in the treatments including the EE suggest that it has hepatoprotective activity, protecting liver cells from damage.

#### 3.4.2. Relative Liver Weight

Relative liver weight (RLW) exhibited no differences between the control and EE groups (Table 1). However, it increased in the CCl_4_ group (*p*<0.001 vs. control), indicating that CCl_4_ induced hypertrophy (hepatomegaly). The Sil + CCl_4_, and EE + CCl_4_ groups did not differ from the control group, and their RLW results were lower than in the CCl_4_ group (*p*<0.001). These results suggest that EE affords protection against liver injury in CCl_4_ fibrosis induction.

#### 3.4.3. Biochemical Markers of Hepatic Injury

Liver injury from introduction of infectious agents or chemicals causes increases in hepatic enzyme serum levels [32].The hepatic enzymes AST, ALT, and AP were employed as biochemical markers of chronic hepatic damage (Table 2).

In the control group AST activity was 85.33 ± 16.03 U/L and ALT activity was 59.00 ± 9.40 U/L; these were considered baseline enzyme activity levels in rats without liver damage. Levels did not differ from the baseline in the EE group. Treatment with CCl_4_ raised AST 3.5-fold over the baseline and AST levels 3.3-fold (*p*<0.001). Cotreatment with silymarin (Sil + CCl_4_ group) produced lesser increases, with a 2.6-fold rise in AST and a 1.8-fold rise ALT (*p *<0.001). In the EE + CCl_4_ group AST levels increased by 3.3-fold and ALT levels by 1.8-fold (*p*<0.001) (Table 2). Similar results were reported by Woong et al. (2004) in a study of* Sargassum henslowianum* and* S. siliquastrum *extracts on CCl_4_-induced hepatotoxicity in male Sprague-Dawley rats in which administration of* S. siliquastrum *methanol crude extract (300 mg/kg) six hours after CCl_4_ treatment was the only treatment to significantly reduce CCl_4_-induced acute elevation of AST and ALT activities [33].

The control group exhibited an AP activity of 120.17 ± 12.67 U/L, which was treated as the baseline level. This did not differ from AP activity in the EE group. In the CCl_4_ group, AP activity was 1.7-fold higher than the baseline (*p*<0.001), and in the Sil + CCl_4_ and EE + CCl_4_ groups it was 1.3-fold higher (*p*<0.01)(Table 2).

Serum levels of ALB in the control group were 2.57 ± 0.14 g/L, which represents 100%. Levels in the EE group did not differ from the control. In the CCl_4_ group ALB was at 85% of the control group (*p*<0.01). Both the Sil + CCl_4_ (91%) and EE + CCl_4_ (98%) treatments effectively reverted any effect of CCl_4_ on ALB levels (*p*<0.01) (Table 2).

Overall the present results agree with a study by Hira et al. (2017), in which administration of* Sargassum ilicifolium*,* S. lanceolatum*, and* S. swartzii *ethanol extracts (200 mg/kg body weight) for fourteen days in a CCl_4_–induced liver intoxication model showed that the* S. ilicifolium* and* S. swartzii *extracts kept hepatic enzymes levels low after CCl_4_-induced injury [34]. This is analogous to the present results although the 50 mg/kg dose used here is much lower than that used in previous reports on other* Sargassum* species.

#### 3.4.4. Histopathology Analysis

**Figure 3:**
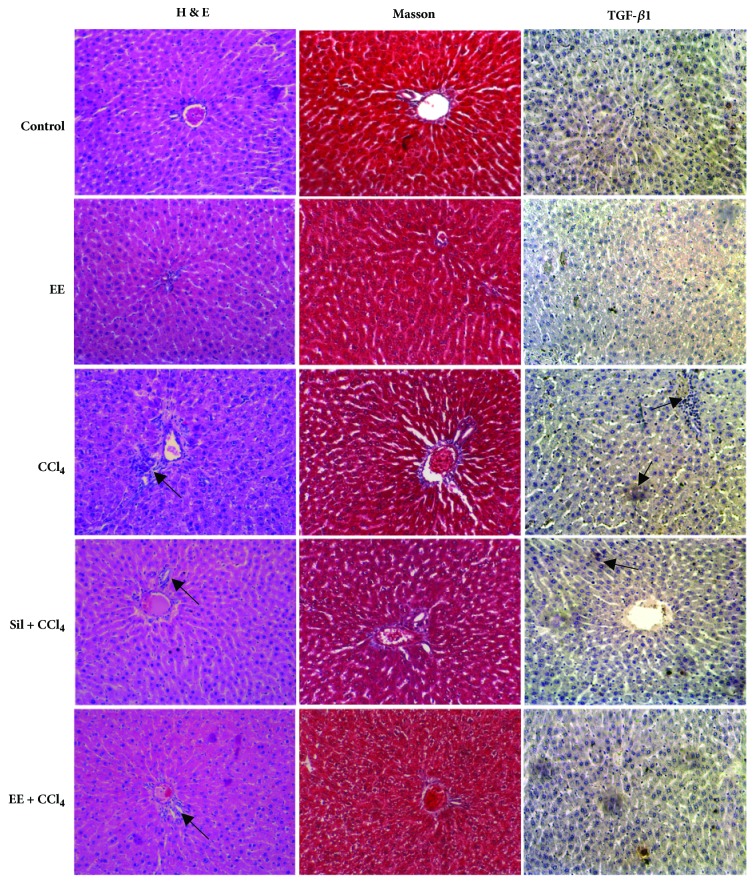
Representative microscopic photographs of rat liver sections stained with hematoxylin and eosin (H&E), Masson's trichrome, and TGF-*β*1 immunohistochemical staining in the experimental groups of a chronic liver damage model in rats. Black arrows in H&E images indicate mononuclear cell infiltration, while in the TGF-*β*1 stain they indicate positive TGF-*β*1 expression.

**H&E staining. **Liver sections from the control group appeared reddish with smooth surfaces, with normal cellular architecture, sinusoidal spaces, and a central vein (Figure 3), which is similar to that observed in the EE group. In contrast, sections from the CCl_4_ group exhibited invasive inflammatory cells and loss of cellular boundaries (Figure 3). Sections from the Sil + CCl_4_ and EE + CCl_4_ groups had a generally normal lobular pattern with a mild degree of lymphocyte infiltration which was near that of the control group (Figure 3). Treatment with EE clearly prevented any notable injury to liver tissues from CCl_4_ exposure.

**Figure 4:**
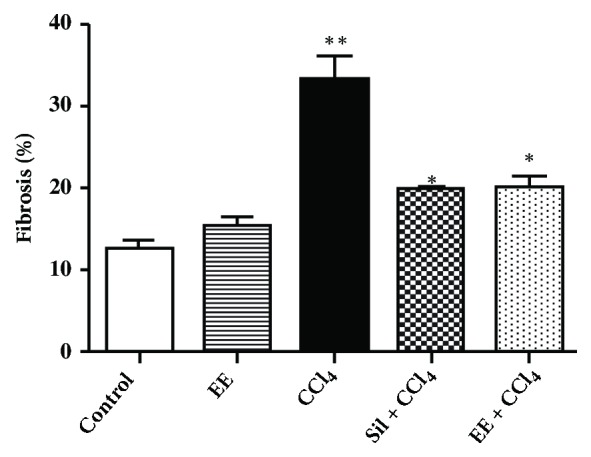
Quantitative fibrosis analysis in the experimental groups of a chronic CCl_4_ liver damage model in rats.* p*-Values: *∗p* < 0.001 vs. CCl_4_, *∗∗* < 0.001 vs. control, according to one-way ANOVA with a Tukey post hoc test.

**Masson trichrome staining**. The control group (Figure 3) showed normal hepatic architecture and no signs of collagen deposition, with similar liver architecture observed in the EE group. Sections from the CCl_4_ group exhibited increased collagen fiber deposition indicating severe fibrosis. Only mild cell inflammatory infiltration and collagen deposition, indicative of minimal fibrosis, were present in the Sil + CCl_4_ and EE + CCl_4_ groups (Figure 3). Quantitative evaluation using the ImageJ software showed a relative increase of fibrosis in the CCl_4_ group versus the control, an effect reverted by treatment with silymarin or EE (Figure 4). The decreases in degree of fibrosis observed in this analysis again suggest that the EE treatment protected liver tissues from progressive fibrosis.

**Figure 5:**
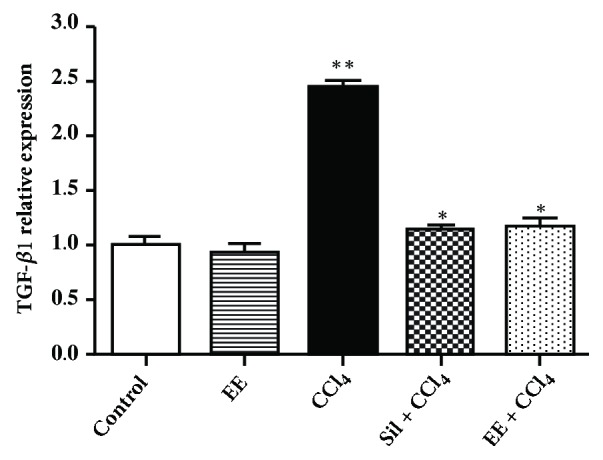
Quantitative immunochemical analysis of TGF-*β*1 expression in liver of the experimental groups in a chronic liver damage model in rats.* p*-Values: *∗* < 0.001 vs. CCl_4_; *∗∗* < 0.001 vs. control, according to one-way ANOVA with a Tukey post hoc test.

**Immunohistochemical analysis**. The cytokine TGF-*β*1 is implicated in hepatic fibrosis. Transforming growth factor beta (TGF-*β*) is produced by numerous inflammatory, mesenchymal, and epithelial cells and converts fibroblasts and other cell types into matrix-producing myofibroblasts. It is expressed in chronic hepatitis, cirrhosis, and cancer [35]. In the control and EE group, expression of TGF-*β*1-positive cells was barely detected, whereas in the CCl_4_ group hepatocytes exhibited significant TGF-*β*1 positive immunoreaction (Figure 3). Expression of TGF-*β*1 was weaker in the Sil + CCl_4_ and EE + CCl_4_ groups (Figure 3) than in the CCl_4_ group (*p*<0.001). Exposure to CCl_4_ clearly induced and increased TGF-*β*1 expression in liver tissue compared to the control, an effect inhibited by treatment with EE and silymarin (Figure 5). In other words, the twelve-week treatment with EE reduced the TGF-*β*1 expression induced by CCl_4_.

One possible approach to preventing, diminishing, and/or reverting fibrosis in the liver is reduction of TGF-*β*1 expression in conditions of chronic liver damage. The EE evaluated here was apparently effective at doing just that, suggesting it may be a potentially promising hepatoprotective agent. This is the first report of an ethanol extract* S. fluitans *exhibiting a hepatoprotective effect* in vivo *in acute and chronic models of liver damage. Ample* S. fluitans* is available along Mexico's Caribbean coast, highlighting the possibility of producing a natural hepatoprotective agent at low cost while mitigating ecological challenges.

## 4. Conclusion

An ethanol extract of the macroalga* Sargassum fluitans* exhibited hepatoprotective effects in both* in vivo *acute and a chronic liver damage models in rodents. Analysis by HPLC found the extract to exhibit major peaks corresponding to phlorotannin dimers and trimers, suggesting these may be involved in the hepatoprotective activity. A very low dose (50 mg/kg) exerted this activity in both the models, although maximum tolerable dose in mice was one hundred times higher (5 g/kg). The acute damage model, using APAP in mice, demonstrated that the extract lowered the enzymatic markers of liver injury induced by APAP. The chronic damage model, using CCl_4_ in rats, confirmed that the extract had a hepatoprotective effect over a twelve-week period, reducing fibrosis and other liver damage and suppressing TGF-*β*1 expression. These rather positive results highlight the promise of the evaluated* S. fluitans *ethanol extract as an element in treatment of liver damage. This macroalga is a plentiful, easily harvested resource that could make a notable contribution to the prevention and treatment of fibrosis. Further research will need to include complete analysis of its composition and potentially active compounds.

## Figures and Tables

**Table 1:** Effect of *S. fluitans *ethanol extract on relative liver weight (RLW), ALT, and AST serum activities in five groups of an acute liver damage model in mice. Results are expressed as means ± SD (n = 4). *p*-Values:  ^&^ < 0.01 vs. APAP; *∗* < 0.01 vs. control; *∗∗* < 0.001 vs. APAP; *∗∗∗* < 0.001 vs. control, according to one-way ANOVA with a Tukey post hoc test.

**Groups**	**RLW **	**ALT (U/L)**	**AST (U/L)**
1. Control	8.37 ± 0.36	44.75 ± 0.41	68.25 ± 0.73
2. EE	8.41 ± 0.23	44.87 ± 0.65	69.54 ± 0.86
3. APAP	9.21± 0.14*∗*	199.50 ± 0.52*∗∗∗*	472.50 ± 0.58*∗∗∗*
4. NAC + APAP	8.39± 0.32^&^	85.75 ± 0.76*∗∗*	311.50 ± 0.49*∗∗*
5. EE + APAP	8.48± 0.13^&^	123.00 ± 0.43*∗∗*	357.00 ± 0.77*∗∗*

**Table 2:** Effect of *S*. *fluitans *ethanol extract on relative liver weight (RLW) and serum biochemical parameters in five groups of a chronic liver damage model in rats. Results are expressed as means ± SD (n = 6). *p*-Values: ^&^<0.01 vs. CCl_4_; *∗*<0.001 vs. CCl_4_; *∗∗*<0.001 vs. control; *∗∗∗*<0.01 vs. control, according to one-way ANOVA with a Tukey post hoc test.

**GROUPs**	**RLW (g)**	**AST (U/L)**	**ALT (U/L)**	**AP(U/L)**	**ALB(g/L)**
1. Control	2.90 ± 0.07	85.33 ± 16.03	59.00 ± 9.40	120.17 ± 12.67	2.57 ± 0.14
2. EE	2.79 ± 0.06	96.33 ± 23.98	71.67 ± 8.71	117.67 ± 11.31	2.55 ± 0.09
3. CCl_4_	3.19 ± 0.08*∗∗*	303.17 ± 38.61*∗∗*	194.17 ± 9.83*∗∗*	213.33 ± 34.48*∗∗*	2.20 ± 0.11*∗∗∗*
4. Sil + CCl_4_	2.87 ± 0.11*∗*	114.50 ± 16.91*∗*	102.50 ± 10.52*∗*	157.00 ± 26.86*∗*	2.36 ± 0.22
5. EE + CCl_4_	2.88 ± 0.11*∗*	90.67 ± 5.68*∗*	103.17 ± 14.84*∗*	157.67 ± 26.75*∗*	2.53 ± 0.15^&^

## Data Availability

The data used to support the findings of this study are available from the corresponding author upon request.

## Supplementary Materials

Supplementary Materials Mass spectrum of peaks identified in an ethanol extract of* Sargassum fluitans* Borgesen at 16.21 min, phlorotannin dimer (A); 18.42 min, isomeric phlorotannin dimer (B); and 19.90 min, phlorotannin trimer (C).
