# Supplementary material for: A Sargassum fluitans Borgesen Ethanol Extract Exhibits a Hepatoprotective Effect In Vivo in Acute and Chronic Liver Damage Models
Source: Biomed Res Int. 2018 Dec 20;2018:6921845. doi: 10.1155/2018/6921845 (PMC6317085; doi:10.1155/2018/6921845)
Supplement: Supplementary Materials — Mass spectrum of peaks identified in an ethanol extract of Sargassum fluitans Borgesen at 16.21 min, phlorotannin dimer (A); 18.42 min, isomeric phlorotannin dimer (B); and 19.90 min, phlorotannin trimer (C). [file 6921845.f1.docx]

**Supplementary material**


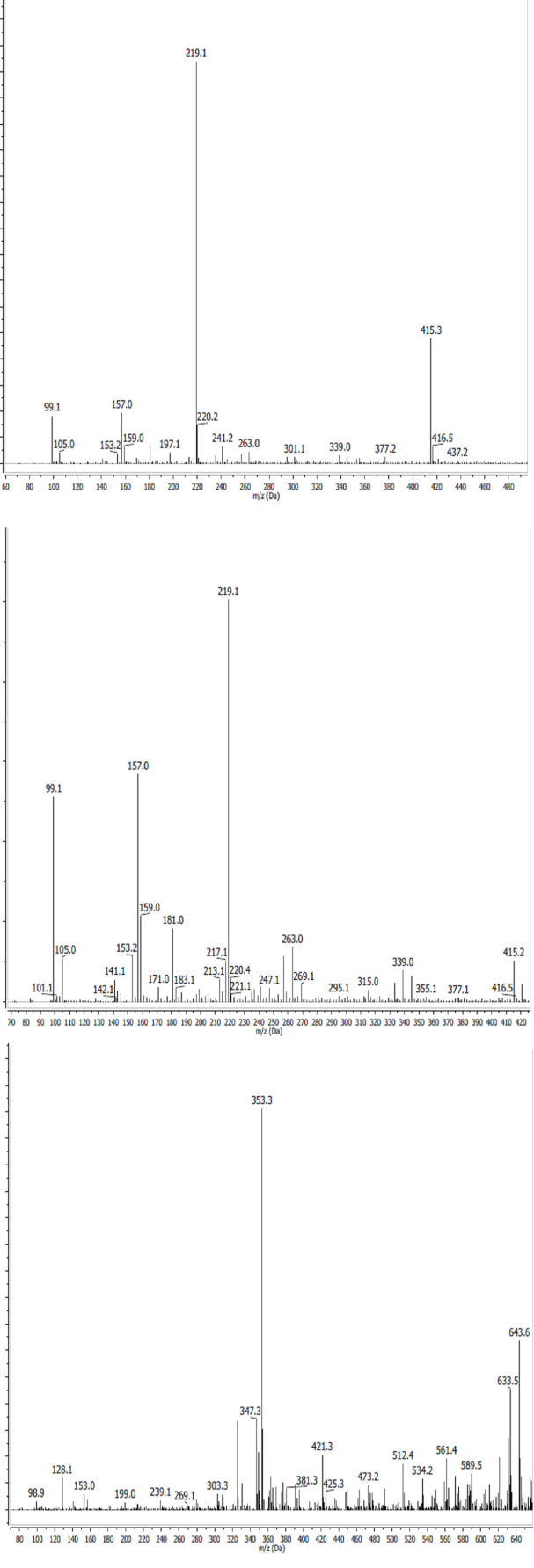


**C**

**B**

**A**

**Figure S1** Mass spectrum of the identified peaks from ethanolic extract of *Sargassum fluitans* Borgesen at 16.21 min (A), 18.42 min (B), and 19.90 min (C).
